# Physicochemical and Antimicrobial Properties of Thermosensitive Chitosan Hydrogel Loaded with Fosfomycin

**DOI:** 10.3390/md19030144

**Published:** 2021-03-06

**Authors:** Luke J. Tucker, Christine S. Grant, Malley A. Gautreaux, Dhanush L. Amarasekara, Nicholas C. Fitzkee, Amol V. Janorkar, Anandavalli Varadarajan, Santanu Kundu, Lauren B. Priddy

**Affiliations:** 1 Department of Agricultural and Biological Engineering, Mississippi State University, Mississippi State, MS 39762, USA; ljt117@msstate.edu (L.J.T.); csg275@msstate.edu (C.S.G.); mag810@msstate.edu (M.A.G.); 2 Department of Chemistry, Mississippi State University, Mississippi State, MS 39762, USA; dla216@msstate.edu (D.L.A.); nfitzkee@chemistry.msstate.edu (N.C.F.); 3 Department of Biomedical Materials Science, University of Mississippi Medical Center, Jackson, MS 39216, USA; ajanorkar@umc.edu; 4 Department of Chemical Engineering, Mississippi State University, Mississippi State, MS 39762, USA; av234@msstate.edu (A.V.); santanukundu@che.msstate.edu (S.K.)

**Keywords:** fosfomycin, chitosan, hydrogel, *S. aureus*, rheology, antimicrobial, physicochemical, thermosensitive, drug delivery

## Abstract

Thermosensitive chitosan hydrogels—renewable, biocompatible materials—have many applications as injectable biomaterials for localized drug delivery in the treatment of a variety of diseases. To combat infections such as *Staphylococcus aureus* osteomyelitis, localized antibiotic delivery would allow for higher doses at the site of infection without the risks associated with traditional antibiotic regimens. Fosfomycin, a small antibiotic in its own class, was loaded into a chitosan hydrogel system with varied beta-glycerol phosphate (β-GP) and fosfomycin (FOS) concentrations. The purpose of this study was to elucidate the interactions between FOS and chitosan hydrogel. The Kirby Bauer assay revealed an unexpected concentration-dependent inhibition of *S. aureus*, with reduced efficacy at the high FOS concentration but only at the low β-GP concentration. No effect of FOS concentration was observed for the planktonic assay. Rheological testing revealed that increasing β-GP concentration increased the storage modulus while decreasing gelation temperature. NMR showed that FOS was removed from the liquid portion of the hydrogel by reaction over 12 h. SEM and FTIR confirmed gels degraded and released organophosphates over 5 days. This work provides insight into the physicochemical interactions between fosfomycin and chitosan hydrogel systems and informs selection of biomaterial components for improving infection treatment.

## 1. Introduction

Chitosan (CH) is the deacetylated derivative of chitin with a degree of deacetylation of at least 50% and is regularly used in the food industry as an edible, renewably sourced food preservative. CH has shown antimicrobial effects against bacterial strains including *Escherichia coli* and *Staphylococcus aureus* (*S. aureus*) [1,2]. CH hydrogels are easily produced by combining acid-solubilized CH with beta-glycerol phosphate (β-GP), the neutralizing, biocompatible salt that makes CH solutions thermosensitive [3]. Once the CH solution is chilled, the β-GP stabilizes the CH through ion and hydrogen bonding [4]. As hydrogen bonds are weak, physiological temperatures (37 °C) are sufficient to break them, allowing the CH to precipitate and forming a hydrogel [4]. The gels can be modified to create a composite material, or loaded with antibiotic or chemotherapy drug, for a variety of applications [5]. 

Osteomyelitis, a bone infection, is difficult to treat with traditional antibiotics alone, a challenge further compounded by the rise in antibiotic resistant bacteria [6], most commonly *S. aureus* [7]. Prolonged systemic (IV) or oral administration of an antibiotic in a high enough concentration to be effective may cause kidney and/or liver toxicity [8]. Localized treatment via a biomaterial carrier would allow for tunable release of drug, keeping the concentration high at the infection site while avoiding systemic toxicity. Combining localized treatment with the use of non-traditional antimicrobials is expected to combat antibiotic resistant bacterial infections more effectively, such as *S. aureus* osteomyelitis.

Fosfomycin (FOS) is a unique antibiotic that has traditionally been used in Europe for uncomplicated urinary tract infections in women [9]. FOS is first internalized by the bacteria, and then it inhibits the function of MurA, which is necessary for the first step in peptidoglycan biosynthesis [10]. Due to the unique mechanism of action, little to no cross resistance with other common antibiotics, such as vancomycin or methicillin, have been observed [11]. Thus, FOS is a good candidate to treat antibiotic-resistant bacteria such as methicillin- or vancomycin-resistant *S. aureus* (MRSA or VRSA). Against both MRSA and methicillin sensitive *S. aureus*, FOS’s minimum inhibitory concentration (MIC) was 6 to 10 times lower than that of vancomycin [6,12]. FOS was also reported to have extensive tissue penetration for reaching infections of the bone tissue compared to other antibiotics, likely owing at least in part to its smaller size [9,13]. 

Due to the relatively limited use of FOS [11,14], the interactions between FOS and CH have yet to be explored. FOS must have an epoxide ring to be biologically active [9], such that when the ring is hydrolyzed, the antibiotic is no longer effective [15]. Ring-opened FOS, also known as FOS impurity A, is structurally similar to β-GP and can be generated in the CH gel system via two mechanisms: hydrolysis or nucleophilic attack by an amine group [16]. Loading the CH hydrogel with additional FOS may function to saturate the amine groups in the CH, overcoming any loss of antimicrobial efficacy due to the conversion of some of the FOS to its inactive, ring-opened form.

The objective of this work was to elucidate the effects of FOS on the physicochemical and antimicrobial properties of CH gels. We *hypothesized*: (i) the CH gel would inactivate some FOS, but that effect would be saturated at 9 mg/mL FOS so that antimicrobial efficacy would not be reduced at 9 mg/mL FOS, (ii) the addition of FOS to CH hydrogels would increase the storage (G′) and loss (G″) moduli and reduce the gelation temperature compared to CH alone, and (iii) the CH would retain FOS in the gel matrix by the reaction between FOS and CH.

## 2. Results

### 2.1. Kirby Bauer Antibacterial Assay

**Figure 1:**
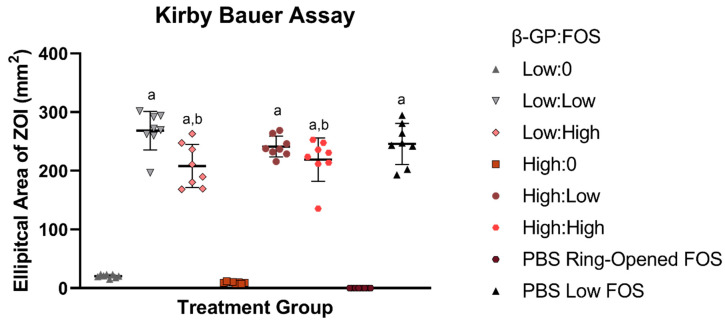
Zones of inhibition (ZOIs) (n = 8) from Kirby Bauer assay after 24-h treatment. Groups with FOS contained 50 µg FOS, except PBS Ring-Opened FOS, which contained 500 µg of ring-opened FOS. All groups containing FOS had larger ZOIs than those without FOS or with ring-opened FOS (^a^ *p* < 0.05). Both groups containing the high concentration of FOS (Low:High and High:High) had smaller ZOIs than the Low:Low group (^b^ *p* < 0.05).

As expected, the addition of FOS increased the zone of inhibition (ZOI), as all groups containing FOS showed enhanced antimicrobial efficacy over those without FOS (Figure 1). None of the CH FOS groups were significantly different from the PBS Low FOS positive control. Interestingly, at the low β-GP concentration, a concentration-dependent effect of FOS was observed, with Low:Low exhibiting a larger ZOI than Low:High while keeping the dose of FOS the same, an effect that was not seen at the high β-GP concentration. Ring-opened FOS exhibited no antimicrobial efficacy, even at a 10× higher dose of FOS than the PBS Low FOS group. The volume of CH gel applied did not significantly affect the ZOI, while the lower β-GP concentration resulted in larger ZOIs (Figure S1).

### 2.2. Planktonic Antibacterial Assay

**Figure 2:**
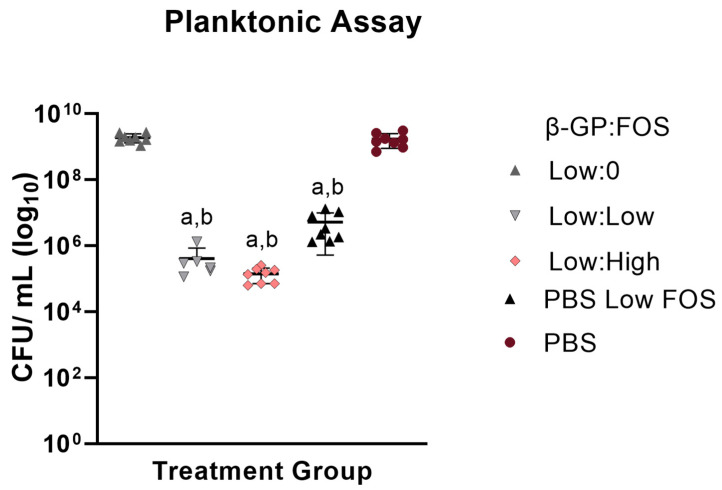
Colony-forming units per milliliter (CFU/mL) of *S. aureus* planktonic populations (n = 6–8) after 24-h treatment. Low:0 and PBS did not contain FOS. Low:Low, Low: High, and PBS Low FOS contained 100 µg FOS. a: significantly lower than Low:0, b: significantly lower than PBS. *p* < 0.05 for all differences indicated.

A planktonic assay was performed to quantify inhibition of *S. aureus* growth in planktonic solution over 24 h. Solutions were supplemented with NaCl to disrupt the CH binding to *S. aureus* [2] and improve quantification of bacterial load. CH alone (Low:0) showed no antimicrobial effect, as it was not different than the PBS only negative control (Figure 2). For both CH gels containing FOS (Low:Low and Low:High), *S. aureus* inhibition was equivalent to the PBS Low FOS positive control (all three groups had the same dose of FOS delivered). The PBS Low FOS showed more killing than the PBS only group. 

### 2.3. Rheology

**Figure 3:**
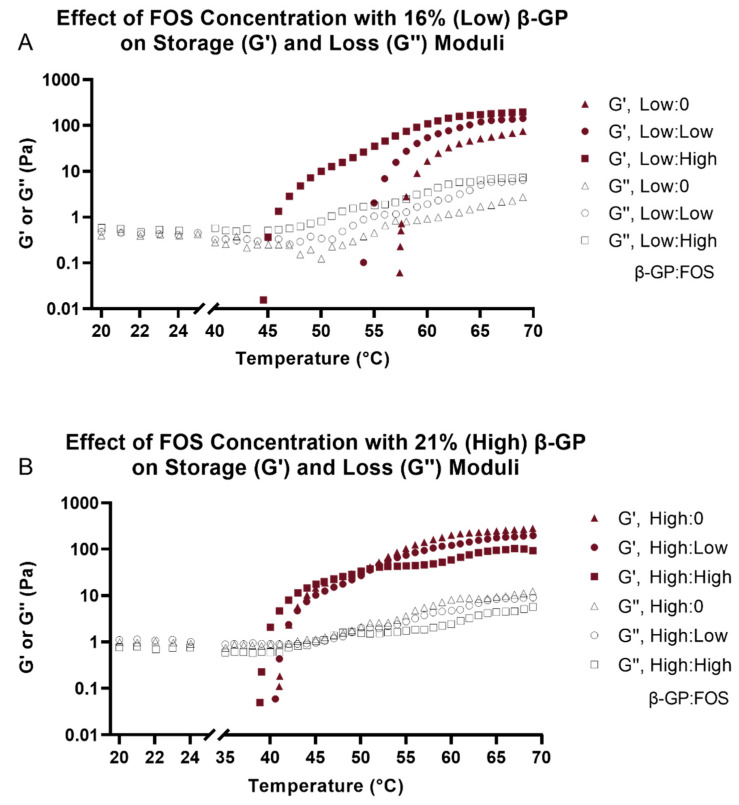
Representative temperature sweep graphs demonstrating storage (G′) and loss (G″) moduli as a function of temperature for 158 mg/mL (Low) β-GP (**A**) and 212 mg/mL (High) β-GP (**B**) with 0, 5, or 9 mg/mL FOS. Geometry gap was 500 µm, temperature ramp was 2℃/min, oscillation frequency was 1 Hz, and strain was 1%.

**Figure 4:**
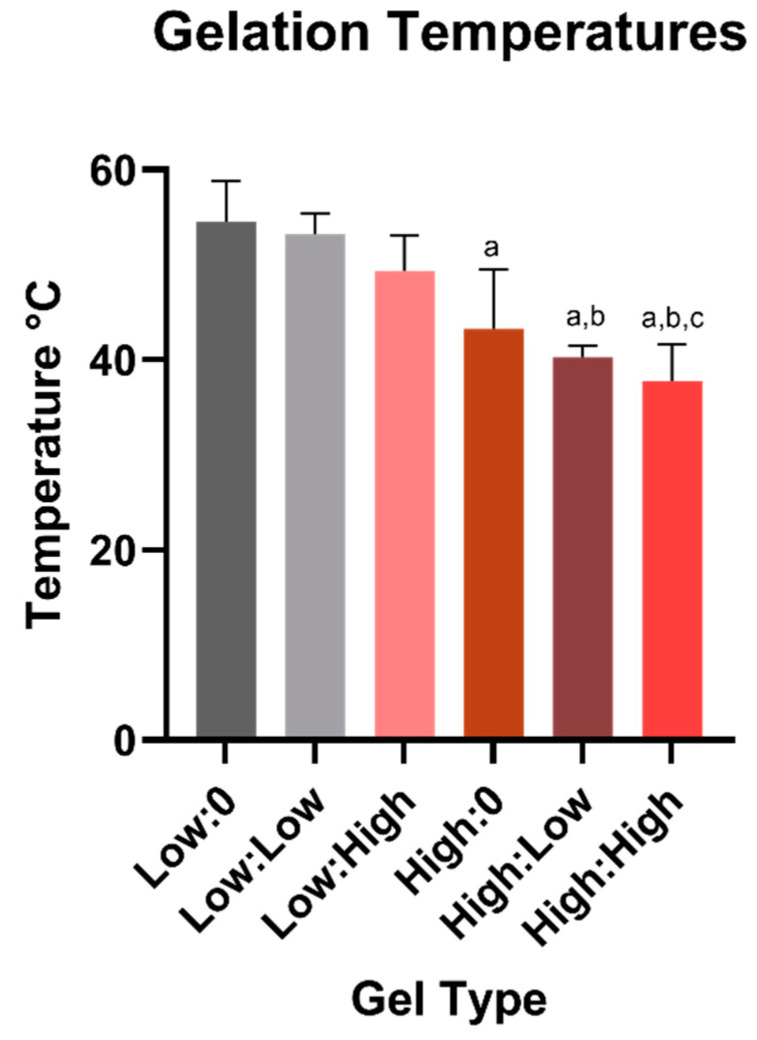
Average gelation temperatures (n = 3) for gels containing 158 mg/mL (Low) β-GP or 212 mg/mL (High) β-GP with 0, 5, or 9 mg/mL FOS (labels are β-GP:FOS). a: significantly lower than Low:0, b: significantly lower than Low:Low, c: significantly lower than Low:High. *p* < 0.05 for all differences indicated.

The storage moduli (G′), loss moduli (G″), and gelation temperature were determined using temperature sweep experiments. The overall rheological responses for all samples were found to be similar. Due to the viscous nature of the samples, initially G′ were very low and could not be measured. However, a rapid increase in G′ was observed once gelation began (Figure 3). Interestingly, the increase in G″ was slower. The crossover between G′ and G″ has been defined as the gelation temperature [17]. In general, as concentration of FOS and β-GP increased, gelation temperature decreased (Figure 4). There was no observed plateau of gelation temperature as a function of β-GP or FOS concentration. 

### 2.4. Scanning Electron Microscopy (SEM)

**Figure 5:**
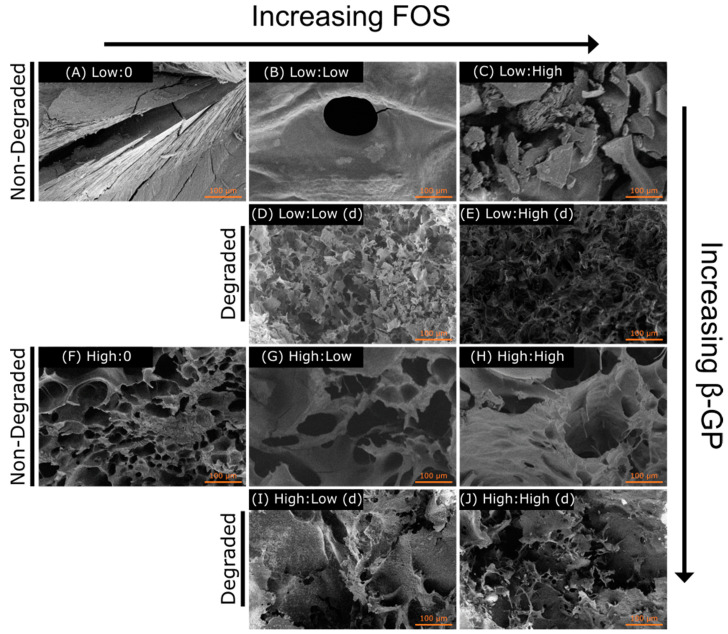
Scanning electron microscopy (SEM) images of freeze-dried CH gel samples. (**A**) 158 mg/mL (Low) β-GP and 0 FOS, (**B**) Low β-GP and 5 mg/mL (Low) FOS, (**C**) Low β-GP and 9 mg/mL (High) FOS, (**D**) degraded Low β-GP and Low FOS, (**E**) degraded Low β-GP and High FOS, (**F**) 212 mg/mL (High) β-GP and 0 FOS, (**G**) High β-GP and Low FOS, (**H**) High β-GP and High FOS, (**I**) degraded High β-GP and Low FOS, and (**J**) degraded High β-GP and High FOS (labels are β-GP:FOS). Samples degraded over 5 days are indicated by (d). Scale bar = 100 µm.

SEM images of most the non-degraded CH hydrogel specimens showed a porous structure, except for the Low:0 group (which appeared non-porous) and the Low:Low group (which appeared noticeably less porous) (Figure 5). All degraded specimens (indicated by d) showed similar open porous structures. The level of porosity and overall porous structure between the degraded specimens and the non-degraded specimens appeared different, with the degraded groups showing more extensive porosity, likely resulting from their degradation in PBS for 5 days.

### 2.5. Nuclear Magnetic Resonance (NMR)

**Figure 6:**
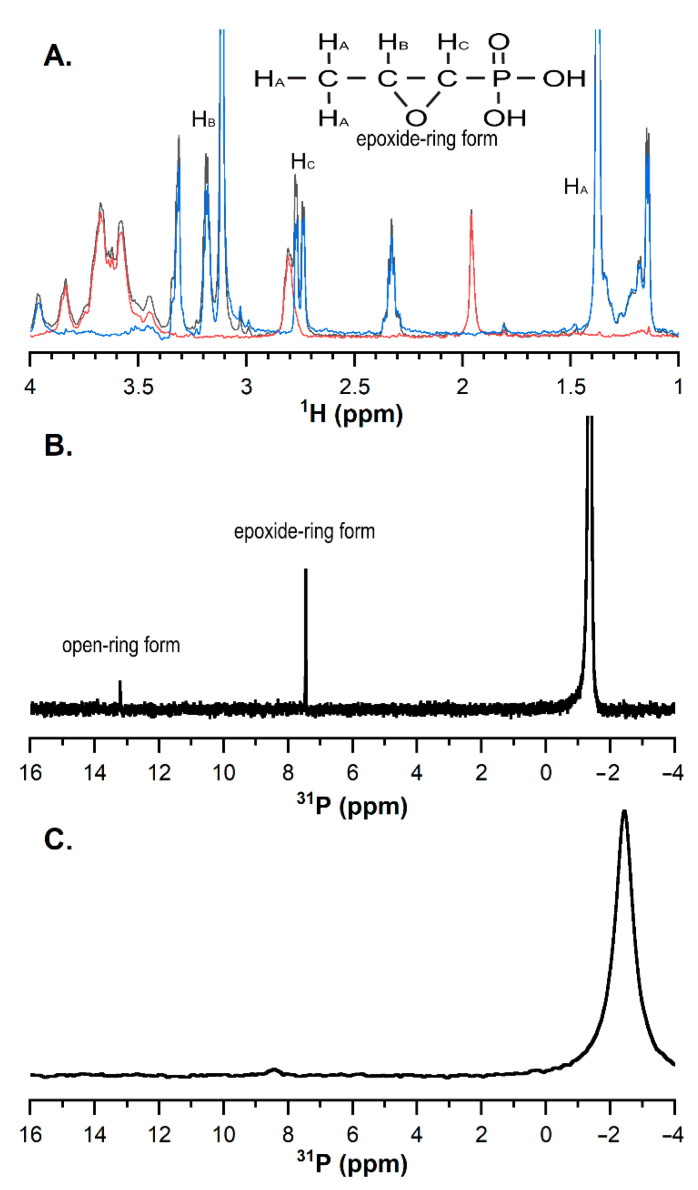
One-dimensional NMR spectra of CH and FOS. (**A**) Comparison of ^1^H NMR spectra of 1 mM FOS (blue), 3 mM CH (red), and a mixture of the two (black) at pH 6.5. This spectrum was unchanged after 6 days. (**B**) ^31^P-NMR spectra of 1 mM FOS with 3 mM CH. No ring-opened FOS (~16 ppm) was observed after 6 days. (**C**) ^31^P-NMR spectrum of 1 mM FOS and 104 mM CH at pH 6.5. The broadened FOS signal was much harder to detect.

At relatively low concentrations, the 1D ^1^H NMR spectra of CH and FOS produced well-resolved and characteristic spectra in the aliphatic region that agrees with Kuzuyama et al. [18] (Figure 6A, red and blue spectra, respectively). When mixing 3 mM of monomeric units/L CH with 1 mM FOS at pH 6.5, some aggregation was observed, but the sample remained a liquid (Figure 6B). This spectrum did not change substantially over 6 days. The 1D ^31^P NMR spectrum showed two major peaks: one large peak, corresponding to the phosphate buffer, and one smaller peak, corresponding to the native, epoxide ring form of FOS (~10 ppm). A small peak corresponding to the ring-opened form is also visible (~13 ppm). 

Adding higher concentrations of CH (104 mM) resulted in a firm gel at pH 6.5. The ^31^P NMR revealed a substantially weaker signal for native (~10 ppm) and ring-opened (~16 ppm) FOS, but a signal for phosphate buffer (~−2 ppm) was still observed (Figure S3). The rotational diffusion of FOS was significantly hindered in the gel, increasing the R_2_ relaxation rate and the corresponding linewidth. Assignments of FOS signals (no CH) are shown in Figure S3. The relative signal intensities from integration (Figure 6B) are comparable to that observed for the sample lacking CH (Figure S3) and peak assignments agree with Jiang et al. [19]. 

**Figure 7:**
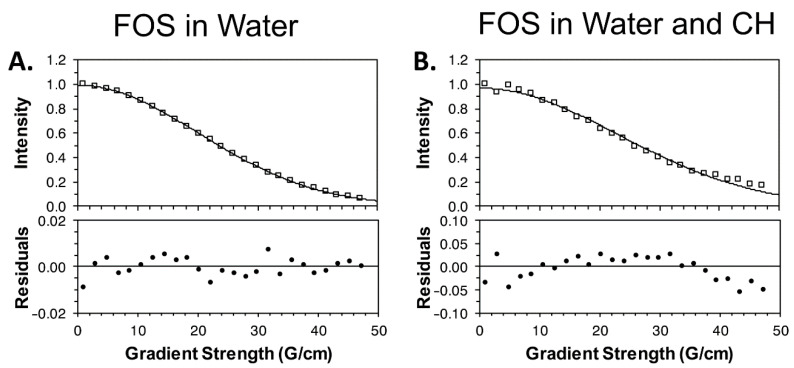
Representative diffusion ordered spectroscopy (DOSY) measurements of FOS in water with D_2_O (**A**) and in the CH matrix (**B**) for the FOS peak at 1.15 ppm. In the upper panel, experimental intensities are shown (open squares) along with the best-fit curve. The lower panels show the fitting residuals. The signal to noise is substantially lower for panel B, resulting in a larger distribution of residuals.

To test the mobility of FOS in the hydrogel, ^1^H diffusion ordered spectroscopy (DOSY) experiments were performed [18,19] in water and in the CH matrix, both with D_2_O. While peaks were very broad and had low signal to noise in the hydrogel, fitting three independent aliphatic FOS peaks yielded an apparent translational diffusion coefficient of (6.2 ± 0.9) × 10^−10^ m^2^ s^−1^, where the uncertainty is the standard error of the mean (Figure 7). This is significantly lower (*p* < 0.01) than the free FOS (with no hydrogel), which has an apparent translational diffusion coefficient of (9.4 ± 0.3) × 10^−10^ m^2^ s^−1^ under the same conditions but without CH.

### 2.6. Fourier-Transform Infrared Spectroscopy (FTIR)

**Figure 8:**
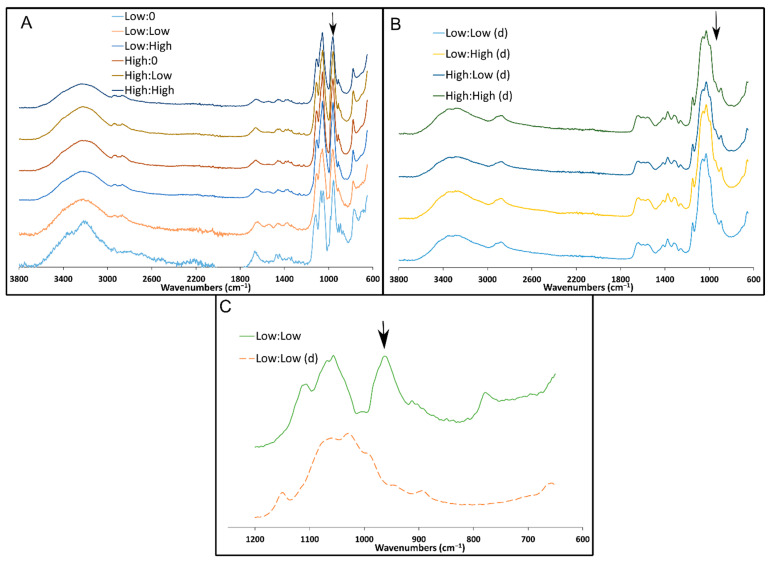
FTIR spectra of freeze-dried CH gel samples containing 158 mg/mL (Low) β-GP or 212 mg/mL (High) β-GP with 0, 5, or 9 mg/mL FOS (labels are β-GP:FOS). (**A**) Non-degraded samples displayed a characteristic peak around 950 cm^−1^ (arrow) indicating FOS and β-GP. (**B**) Samples following 5 days of degradation (indicated by d). (**C**) Subset (1200 to 650 cm^−1^) of representative non-degraded (solid line) and degraded (dashed line) spectra. The degraded spectrum lacked the peak around 950 cm^−1^ (arrow), suggesting the release of FOS and β-GP.

The FTIR spectra showed that all non-degraded specimens were similar in composition, with characteristic peaks around 950, 1050, 1110, 1350, 1425, 1645, 2850, 2900, and 3200 cm^−1^ (Figure 8A). Multiple peaks representing the chitosan matrix can be seen in the FTIR spectra [20,21,22]. The peak around 1050 cm^−1^ indicates C-O stretching vibrations. The peak around 1110 cm^−1^ indicates C-O-C stretching vibrations. The peaks around 1350 and 1645 cm^−1^ indicate the amide-III peak representing the N-H stretching vibrations and amide-I peak representing the C=O stretching vibrations, respectively. The peaks around 1425, 2850, and 2900 cm^−1^ indicate the C-H vibrations. The peak around 3200 cm^−1^ indicates the N-H and O-H stretching vibrations. Additionally, the peaks around 950 and 1110 cm^−1^ indicate P-O stretching vibrations, and the shoulder from ~1070 cm^−1^ to 1050 cm^−1^ indicates stretching vibrations in PO_3_^2−^ in the FOS [23] as well as in the β-GP [24].

The FTIR spectra for all degraded specimens (degraded in PBS for 5 days) had similar chemical composition and peaks representing the chitosan matrix as those observed for the non-degraded specimens (Figure 8B). One striking difference in the FTIR spectra for the degraded specimens versus those for the non-degraded specimens was the loss/reduction in the peak around 950 cm^−1^ (Figure 8C), suggesting the release of the FOS from the hydrogels.

## 3. Discussion

It has been previously established that CH and FOS are effective in inhibiting *S. aureus* growth independently [2,9]. Here, FOS had a positive effect on the antimicrobial properties of CH hydrogels, as demonstrated by the decrease in bacterial growth in both the planktonic assay and the Kirby Bauer assay. In the Kirby Bauer assay, antimicrobial must first be released from its delivery vehicle, traditionally saturated paper disks, but here, the samples were pipetted directly onto the agar plate. Then, the antibiotic must diffuse through the agar to reach bacteria and prevent growth, which is likely why increasing the volume of CH did not increase the ZOI (Figure S1). While keeping the dose of FOS constant, an effect of FOS concentration was expected, whereby the high FOS concentration groups (Low:High and High:High) were expected to have larger ZOIs compared to their lower concentration FOS counterparts (Low:Low and High:Low, respectively). Surprisingly, the converse was observed, but only at the low β-GP concentration. In other words, the ZOI for Low:Low was greater than that for Low:High, likely due to differences in release/availability of the antibiotic from the gel as a function of gel stiffness. This effect was not observed at the high β-GP concentration, likely due to the decrease in ZOI from reduced spreading of the high β-GP concentration (FOS-free) gels on the agar, regardless of volume delivered (Figure S1). It was noted that in placing the gel onto the agar plate, the low β-GP groups spread out more, suggesting a lower modulus. This effect of β-GP on physical properties was quantified via rheology and may explain the higher antimicrobial efficacy with the low β-GP concentration (compared to high β-GP) in the absence of FOS (Figure S1). As differences were observed between the low β-GP (but not between the high β-GP) concentration groups in the Kirby Bauer assay, only the low β-GP concentration groups were evaluated in the planktonic assay.

*S. aureus* has a planktonic stage in its pathogenesis [25]; therefore, of the two assays, the planktonic assay served to more closely mimic *S. aureus* pathogenesis in vivo. Due to the fluid environment of the assay, the diffusivity of the material was believed to have less of an effect (relative to the Kirby Bauer assay) on the inhibition of the bacteria. Despite no antimicrobial effect of the CH itself, notably, the bacterial colonies were smaller for the CH groups compared to those for the PBS groups. This could be due to CH’s ability to act as a flocculant [26], a chemical that induces particle sedimentation. To minimize the flocculant effect of CH [2] and reduce the risk of this effect impeding accurate quantification of bacterial loads, NaCl was added to the planktonic solution. At the low NaCl concentration used (5% *w/v*), no effect of NaCl on bacterial growth was observed (Figure S2). Though minimal, ZOIs for groups without FOS were nonzero, indicating chitosan’s innate antimicrobial inhibition of *S. aureus*, an enhanced functionality other hydrogel systems such as poloxamer 407, which did not offer any antimicrobial activity [27]. Future biological testing will include antibiofilm assays to challenge the CH biomaterial system with the difficult to treat biofilm stage of *S. aureus* pathogenesis.

Evaluation of the physical properties of CH hydrogels showed that both FOS and β-GP influenced gelation temperature, storage modulus (G′), and porosity. Similar increases in G′ and G′′ with higher β-GP concentrations have been observed previously [28]. Although differences in G′ and G′′ were more apparent at the low β-GP concentration, the effects of FOS were not negligible at the high β-GP concentration. It has been shown that increasing β-GP concentration can mask the effect of molecular weight on gel rheology [29], suggesting the effect of β-GP concentration may have overpowered the effect of FOS concentration on moduli observed here. There was a reduction in gelation temperature with increasing β-GP concentration, an effect that was observed for all concentrations of FOS. Future rheological testing will include frequency and amplitude sweeps to further understand the interaction of CH and FOS on gel mechanics. The SEM images supported the rheological findings, whereby a more porous structure in the CH matrix was observed with increasing β-GP and FOS concentrations. This agrees with prior results that demonstrated increasing the salt composition decreased the homogeneity of the hydrogel density profile, thus increasing the porosity of the freeze-dried architecture [28]. Overall, the porous structure of these scaffolds indicates their suitability for drug delivery and tissue engineering applications by allowing for cell infiltration and biomolecule delivery. Sample architecture was altered following degradation in PBS for 5 days, most likely due to the low molecular weight of the CH allowing the monomer and oligomer particles to be suspended in solution and the change in concentration of salts during degradation. Mass loss over time should be measured to predict the longevity of the material in vivo.

Finally, the chemical testing confirmed that, during hydrogel formation, some of the FOS is taken out of the liquid phase of the hydrogel and rendered chemically unavailable. Quantifying signals in ^31^P spectra is challenging because of nuclear Overhauser enhancement that occurs during decoupling, but a clear peak was observed for the native FOS in the presence of a low concentration (3 mM) of CH (Figure 6B). This experiment established a baseline FOS signal for comparing samples with higher amounts of CH. The 1D ^1^H and ^31^P NMR spectra of FOS did not change over at least 6 days at low concentrations of CH, and at low concentrations of CH, no reactions appeared to occur. Hydrogel formation at the higher CH concentration complicated NMR measurements because the sample largely behaved like a solid. Moreover, the integral of the FOS signal relative to the phosphate buffer decreased over time, as a 5% reduction was seen over 12 h, supporting the hypothesis that FOS and CH are reacting. FOS and CH reactions would be expected if the FOS reacted with any of the large number of amine groups in the high-concentration CH sample, forming a covalent bond as a result of the nucleophilic attack and epoxide ring opening [16]. Under these circumstances, FOS would adopt the rotational diffusion time of the CH hydrogel, leading to a fast R_2_ relaxation rate and extremely broad peaks [30]. Though challenges with the hydrogel sample preclude an absolute quantitation of ^31^P signal originating from FOS, the decrease in intensity from the P^31^ NMR spectra relative to the phosphate peak suggests that over time, FOS became immobilized in the CH matrix, rendering it chemically unavailable, as confirmed with the lower apparent translational diffusivity coefficient compared to CH alone. Thus, a combination of factors is likely at play to immobilize FOS in the CH matrix, including chemical reactivity with the CH matrix itself, and slowed translational and rotational diffusion within the matrix. The slowed diffusion is likely mediated by hydrogen bonding and other non-covalent interactions between the FOS and the CH matrix. FTIR of gels after 5 days of degradation revealed the FOS and β-GP signals decreased in strength, suggesting the FOS and β-GP were released as the CH gels degraded. Additional experiments including solid phase extraction of the chitosan degradation products for quantification of released FOS by liquid chromatography-mass spectrometry (LC-MS) are needed to fully understand the specific chemical interactions between FOS and CH. Due to fosfomycin’s optical inactivity, direct quantification is difficult to achieve. A method for quantification via LC-MS has been reported [31]. From a preliminary experiment using these methods, it is believed the low molecular weight chitosan (degradation products, i.e., chitosan monomer/oligomer) used here may have interfered with the hydrophilic interaction liquid chromatography column, causing retention time peaks of the eluent to broaden and vary drastically compared to the standard curve samples, preventing accurate quantification of the released FOS.

## 4. Materials and Methods 

### 4.1. Materials

Low molecular weight CH with degree of deacetylation of 95.5%, viscosity of 39 cps 1% CH in 1% acetic acid and sourced from blue water crab shell from China (information provided by the supplier) was used for all experiments (Sigma-Aldrich, #448869, Saint Louis, MO, USA). Phosphate buffered saline (PBS) prepared from tablets (Sigma-Aldrich, P4417, Saint Louis, MO, USA) was used for all experiments except for the gel degradation experiment, in which PBS with calcium and magnesium ions was used (ThermoFisher, #14040-117, Waltham, MA, USA). Fosfomycin (FOS) disodium salt was sourced from MP Biomedicals, LLC, #151876 (Santa Ana, CA, USA). Beta-glycerol phosphate (β-GP) was sourced from EMD Millipore Corp, #35675 (Temecula, CA, USA). Hydrochloric acid (1 M) was sourced from Supelco Titripur®, #1.09057.1000 (Kenilworth, NY, USA). Brain Heart Infusion (BHI) #211059 and Difco^TM^ Agar Bacteriological #214530 were purchased from BD (Franklin Lakes, NJ, USA). Sodium chloride was purchased from Sigma-Aldrich (Saint Louis, MO, USA).

### 4.2. Chitosan Gel Creation

Two hundred mg of CH was measured into a 20 mL glass scintillation vial. Nine mL of 0.1 M HCl was slowly added and constantly stirred for 3 h. In a separate tube, 1 mL of water was added to 1.12 g of β-GP and vortexed until homogenous. Both the CH and β-GP solutions were autoclaved at 121°C for 30 min, cooled to room temperature, and placed on ice. While the CH solution was stirring in the ice bath, either 1.88 or 1.12 g of β-GP powder was slowly added to the CH solution. Once the solution was homogeneous, 1 mL of the β-GP solution was added dropwise into the CH solution to create either 156 or 212 mg/mL *w/v* β-GP concentrations. The solutions were stored at 4 °C up to one month. The FOS was added by resuspending 69.4 or 125 mg in 1 mL PBS, then taking 36 μL of the solution and adding it to 464 μL of the CH + β-GP solution in a single 1 mL Luer Lock syringe. The solutions were mixed by a dual-syringe system. The syringes were stored at 4 °C for up to 24 h before being used. The FOS solution was stored at −80 °C for up to 6 months.

### 4.3. Kirby Bauer Antibacterial Assay

A modified Kirby Bauer assay was performed as previously reported [6]. A colony of *S. aureus* (ATCC 6538-GFP) was grown overnight at 37 °C with shaking (150 rpm) for 18 h. A sterile cotton swab was used to spread the culture onto BHI agar plates. Then, the treatments were directly pipetted onto the plates (without the use of disks as traditionally performed). All nine treatment groups outlined in Table 1 were evaluated. An additional four treatments (Table S1) were evaluated to determine whether volume of chitosan affected ZOI. The plates were incubated statically for 24 h at 37 °C. The elliptical zone of inhibition (ZOI) was calculated by measuring the major and minor diameters (d1 and d2, respectively) from the equation for the area of an ellipse: ZOI = π(d1/2) ∗ (d2/2).

### 4.4. Planktonic Antibacterial Assay

This assay was modified from a previous planktonic antibacterial assay [27] to include sodium chloride in the growth medium. Briefly, a colony of *S. aureus* modified to express green fluorescent protein (ATCC 6538-GFP) [6] was grown in 4 mL of Brain Heart Infusion (BHI) media overnight at 37 °C with 150 rpm shaking. Aliquots (200 µL each) of the overnight culture were added to five culture tubes containing 4 mL of BHI. These cultures were grown to an optical density (OD) at 600 nm of 0.2 (µQuant, BioTek Instruments, Inc., Winooski, VT, USA) [32]. The cultures were centrifuged at 4000× *g* rpm for 4 min; then 20 mL of culture were resuspended in fresh BHI containing 1% glucose (*w/v*) and 5% NaCl (*w/v*). Then, 500 μL of culture in BHI with glucose and NaCl was pipetted into non-tissue culture treated 24-well plates. Five treatment groups (n = 6–8) were investigated: low β-GP CH gel groups containing 0, 5, or 9 mg/mL FOS; PBS with 5 mg/mL FOS; PBS. A control group of PBS with no additional NaCl was used to determine the effects of NaCl on bacterial growth. For groups containing FOS, twice the volume listed in Table 1 was delivered (indicated by *), to double the dose of FOS compared to the dose used in the Kirby Bauer assay. Treated cultures were incubated at 37 °C with shaking at 150 rpm for 24 h. Solutions were collected, serially diluted, and plated for bacterial counting.

### 4.5. Rheology

Rheological experiments were conducted using a TA Instruments Discovery HR-2 Hybrid Rheometer (TA Instruments, New Castle, DE, USA). Then, 20 mm parallel plate geometry with a solvent trap was used and the experimental temperature was controlled using a Peltier system. Six groups were evaluated in triplicate: 158 mg/mL (Low) β-GP or 212 mg/mL (High) β-GP with 0, 5, or 9 mg/mL FOS. Samples were prepared using the dual-syringe system and ejected onto the bottom plate of the rheometer maintained at 20 °C. The samples were transported in an icebox and stored there until loaded into the rheometer. The top plate was lowered to 500 µm, and the excess material was wiped away. Samples were subjected to a two-minute soak time to reach thermal equilibrium. A temperature sweep oscillation experiment was conducted with 2 °C/min ramp from 20 to 70 °C with a strain of 1% and a frequency of 1 Hz. The intersection between the storage and loss moduli was considered the gelation temperature.

### 4.6. Chitosan Gel Degradation and Freeze-Drying

The same 6 groups from rheology were evaluated in a degradation experiment: 158 mg/mL (Low) β-GP or 212 mg/mL (High) β-GP with 0, 5, or 9 mg/mL FOS. Then, 100 μL of each gel was pipetted into a 48-well plate, submerged in 700 μL of PBS (with calcium and magnesium ions), and placed on a shaker plate set to 100 rpm in a 37 °C incubator. Solutions were collected and replaced at 6, 12, 24, 48, 72, 96, and 120 h. Then, the gel was collected by carefully scoping from the bottom of the well. Degraded and non-degraded (fresh) samples were placed in microcentrifuge tubes, frozen in a −80 °C freezer overnight, and freeze dried (Labconco FreeZone^1^, Labconco, Kansas City, MO, USA).

### 4.7. Scanning Electron Microscopy (SEM)

The morphology of the freeze-dried samples (n = 3) was accessed by scanning electron microscopy (SEM). A SUPRA 40 scanning electron microscope (Carl Zeiss, Thornwood, NY, USA) was used. The samples were sputter-coated with Au/Pd prior to scanning and were scanned at 5 kV. SEM images were captured at 500× magnification to reveal the key morphological features.

### 4.8. Nuclear Magnetic Resonance (NMR) 

All the experiments were carried out using Bruker Avance (San Jose, CA, USA) III QCI cryoprobe-equipped NMR spectrophotometer operating at a ^1^H frequency of 600 MHz. To observe the interactions between FOS and CH, several NMR samples were prepared, using low (3 mM) and high (104 mM) concentrations of CH, either with or without 1 mM FOS. No samples contained β-GP as it would further complicate ^31^P NMR spectrograms. Additionally, all samples contained 5 % D_2_O and 50 mM sodium phosphate buffer. For the combined FOS and CH samples, the pH was 6.5, and the pH was confirmed after mixing. All samples were prepared as 600 μL solutions before being transferred to NMR tubes. Each ^1^H-NMR spectrum was recorded at 298 K with 128 scans using a perfect-echo WATERGATE sequence to refocus scalar couplings and to suppress solvent [33]. Each ^31^P-NMR spectrum was recorded at 298 K using an inverse-gated decoupling pulse sequence, with 128 scans, a spectral width of 38 ppm, an acquisition time of 1 s, and a recycle delay of 4 s. The native and ring-opened ^31^P signals of FOS were assigned by monitoring the NMR spectra of a pH 2.5 sample by adding 1 M HCl to lower the pH. Diffusion ordered spectroscopy (DOSY) experiments were performed in triplicate, with samples taken from independent peaks in the spectra, and recorded as an interleaved set of 25 experiments, with gradient powers ranging from 1–47 G/cm [18,19]. Water suppression in DOSY experiments was obtained with 4 s of presaturation during the recycle delay. For the free FOS (without CH) DOSY experiments, the diffusion and gradient encoding times (Δ and δ) were 120 ms and 1.3 ms, respectively. For the FOS in CH matrix DOSY sample, Δ and δ were 85 ms and 1.9 ms. The apparent translational diffusion coefficient was found using the Stejskal-Tanner equation. The hydrogel DOSY sample required 512 scans for each gradient strength (approximately 15 h total acquisition time) to obtain sufficient signal to noise for fitting the broadened FOS peaks. 

### 4.9. Fourier-Transform Infrared Spectroscopy (FTIR)

The chemical composition of the freeze-dried samples (n = 3) was assessed by attenuated total reflectance Fourier transform infrared (ATR-FTIR) spectroscopy, and a representative sample was chosen for each group. A Spectrum 100 FTIR spectrophotometer (PerkinElmer, Waltham, MA, USA) was used. Spectra were collected from 600 to 4000 cm^−1^ at 4 cm^−1^ resolution and averaged between four scans.

### 4.10. Statistical Analyses

Data from the Kirby Bauer and planktonic assays, as well as gelation temperature, were analyzed with one-way analysis of variance (ANOVA) with Tukey’s multiple comparisons test with alpha = 0.05, except for the planktonic assay of PBS with and without NaCl, which used a Student’s *t*-test with alpha = 0.05. All aforementioned data are presented as mean ± standard deviation. The apparent translational diffusion coefficients from DOSY were analyzed using a Student’s *t*-test with alpha = 0.01 and are presented as mean ± standard error of the mean.

## 5. Conclusions

Thermosensitive CH hydrogels combined with FOS antibiotic can be advantageous for local antibacterial efficacy. In our antimicrobial assays, the only effect of β-GP or FOS concentration observed was an (unexpected) reduction in killing at the higher FOS concentration, likely the result of entrapment of some FOS within the gel. The effect whereby increasing FOS concentration increased storage (and loss) modulus was more apparent at the lower β-GP concentration, compared to minimal differences in moduli as a function of FOS at the higher β-GP concentration. An increase in β-GP (but not FOS) decreased the gelation temperature of the CH gels. A more porous microstructure was observed with increases in both β-GP and FOS. NMR confirmed the slight reaction of FOS with CH, and that CH significantly slowed the diffusion of FOS compared to FOS in solution. Finally, FOS and β-GP were removed from CH during degradation, as measured by FTIR. Collectively, FOS loaded CH hydrogels demonstrated physicochemical and antimicrobial traits beneficial for infection control in vivo.

## Figures and Tables

**Table 1:** List of treatment groups for antibacterial assays, with concentration of components and volume used.

Treatment Group	CH [mg/mL]	β-GP [mg/mL]	FOS [mg/mL]	Volume [μL]
Low:0	18.6	158	0	10 *
Low:Low	18.6	158	5	10 *
Low:High	18.6	158	9	5.56 *
High:0	18.6	212	0	10
High:Low	18.6	212	5	10
High:High	18.6	212	9	5.56
PBS Low FOS	0	0	5	10 *
PBS Ring-Opened FOS	0	0	5 (ring-opened)	10
PBS	0	0	0	10 *

CH: chitosan, β-GP: beta-glycerol phosphate, FOS: fosfomycin. * Groups had twice the volume delivered for the planktonic assay. Zero percent CH means the FOS was suspended in PBS.

## Data Availability

Data is contained within the supplementary materials.

## Supplementary Materials

The following are available online at https://www.mdpi.com/1660-3397/19/3/144/s1, Table S1: Treatment groups for Kirby Bauer chitosan volume assay, Figure S1: Effects of chitosan volume in Kirby Bauer assay, Figure S2: Effects of NaCl in planktonic assay, Figure S3: 31P acid hydrolysis of fosfomycin
