# Supplementary material for: Physicochemical and Antimicrobial Properties of Thermosensitive Chitosan Hydrogel Loaded with Fosfomycin
Source: Mar Drugs. 2021 Mar 6;19(3):144. doi: 10.3390/md19030144 (PMC8001123; doi:10.3390/md19030144)
Supplement: Supplementary file 1 [file marinedrugs-19-00144-s001.zip › Supplemental/Figure_captions.docx]

**Supplemental Table 1.** List of treatment groups for Kirby Bauer chitosan volume assay, with concentration of components and volume used.

| **Treatment Group** | **CH [mg/mL]** | **β-GP [mg/mL]** | **FOS [mg/mL]** | **Volume [μL]** |
| --- | --- | --- | --- | --- |
| Low:Low V, 0 FOS | 18.6 | 158 | 0 | 5.56 |
| Low:High V, 0 FOS | 18.6 | 158 | 0 | 10 |
| High:Low V, 0 FOS | 18.6 | 212 | 0 | 5.56 |
| High:High V, 0 FOS | 18.6 | 212 | 0 | 10 |

CH: chitosan, β-GP: beta-glycerol phosphate, FOS: fosfomycin.


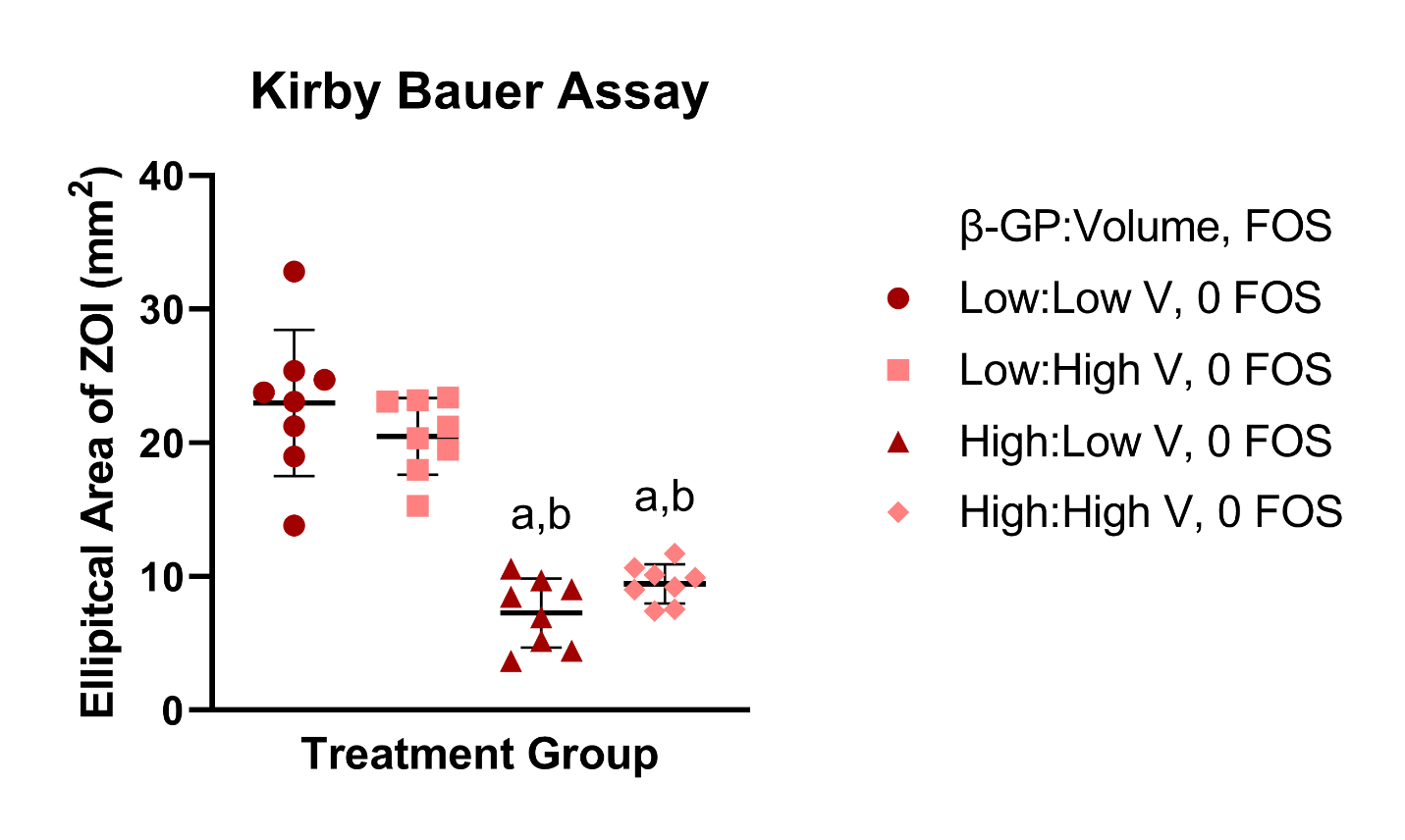


**Supplemental Figure 1.** Zones of inhibition (ZOIs) (n=8) from Kirby Bauer assay after 24-hour treatment with either 5.56 µL (Low) or 10 µL (High) volumes of CH, containing either 158 mg/mL (Low) β-GP or 212 mg/mL (High) β-GP concentrations. No FOS was used in this assay (indicated by 0 FOS). Both groups containing the high β-GP concentration had smaller ZOIs than both lower β-GP concentration groups. ^a^p<0.05 compared to Low:Low V, 0 FOS; ^b^p<0.05 compared to Low:High V, 0 FOS. No effect of CH volume was observed.

**
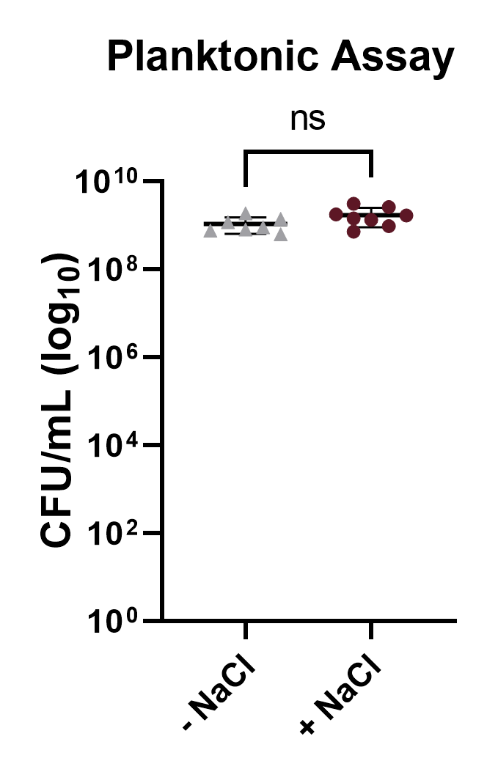
**

**Supplemental Figure 2**. Colony-forming units per milliliter (CFU/mL) of *S. aureus* planktonic populations (n=7-8) after 24-hour treatment with PBS with or without additional 50 mg/mL NaCl. No FOS was used in this assay. ns: not significant at α=0.05.

**
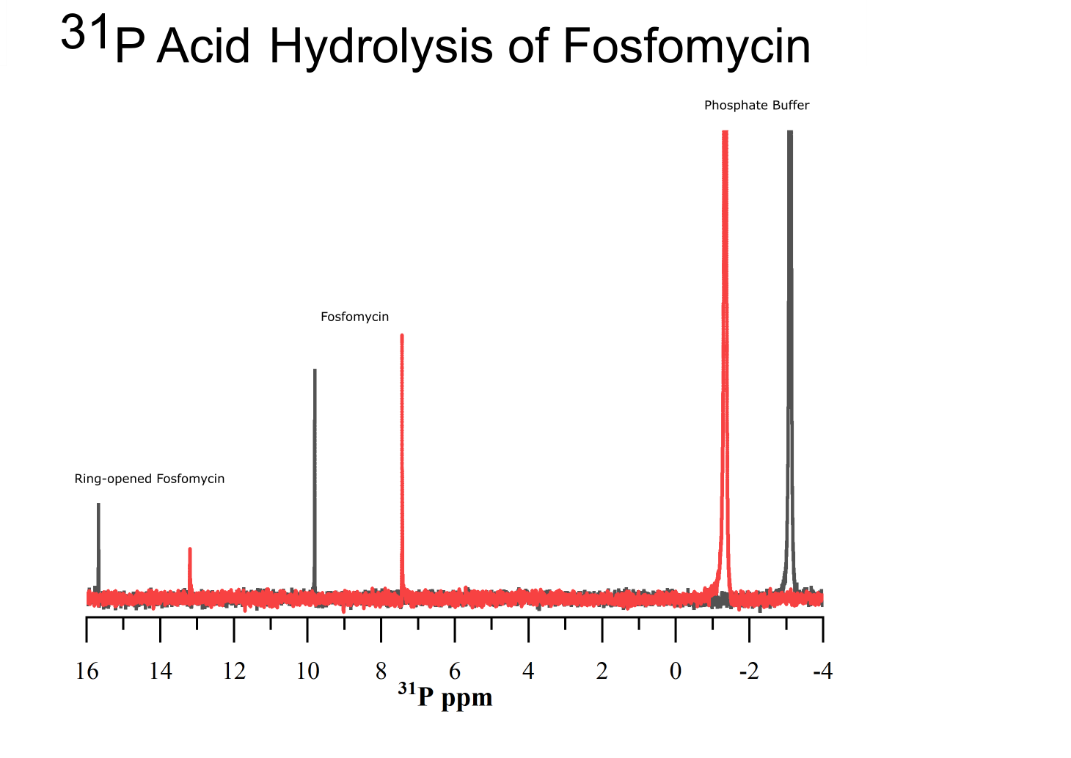
**

**Supplemental Figure 3.** Assignment of the native and ring-opened forms of FOS. A 1D ^31^P spectrum was obtained immediately following preparation of FOS with no CH at pH 2.5. The original spectrum (red) shows two peaks for FOS, an upfield peak corresponding to the epoxide ring form and a downfield peak corresponding to the ring-opened form. After 18 h, the signal corresponding to the ring-opened form had increased relative to the native signal (black spectrum). For clarity, the spectrum recorded at 18 h has been shifted downfield by 1.5 ppm.
