# Supplementary figures and images for: Physicochemical and Antimicrobial Properties of Thermosensitive Chitosan Hydrogel Loaded with Fosfomycin
Source: Mar Drugs. 2021 Mar 6;19(3):144. doi: 10.3390/md19030144 (PMC8001123; doi:10.3390/md19030144)

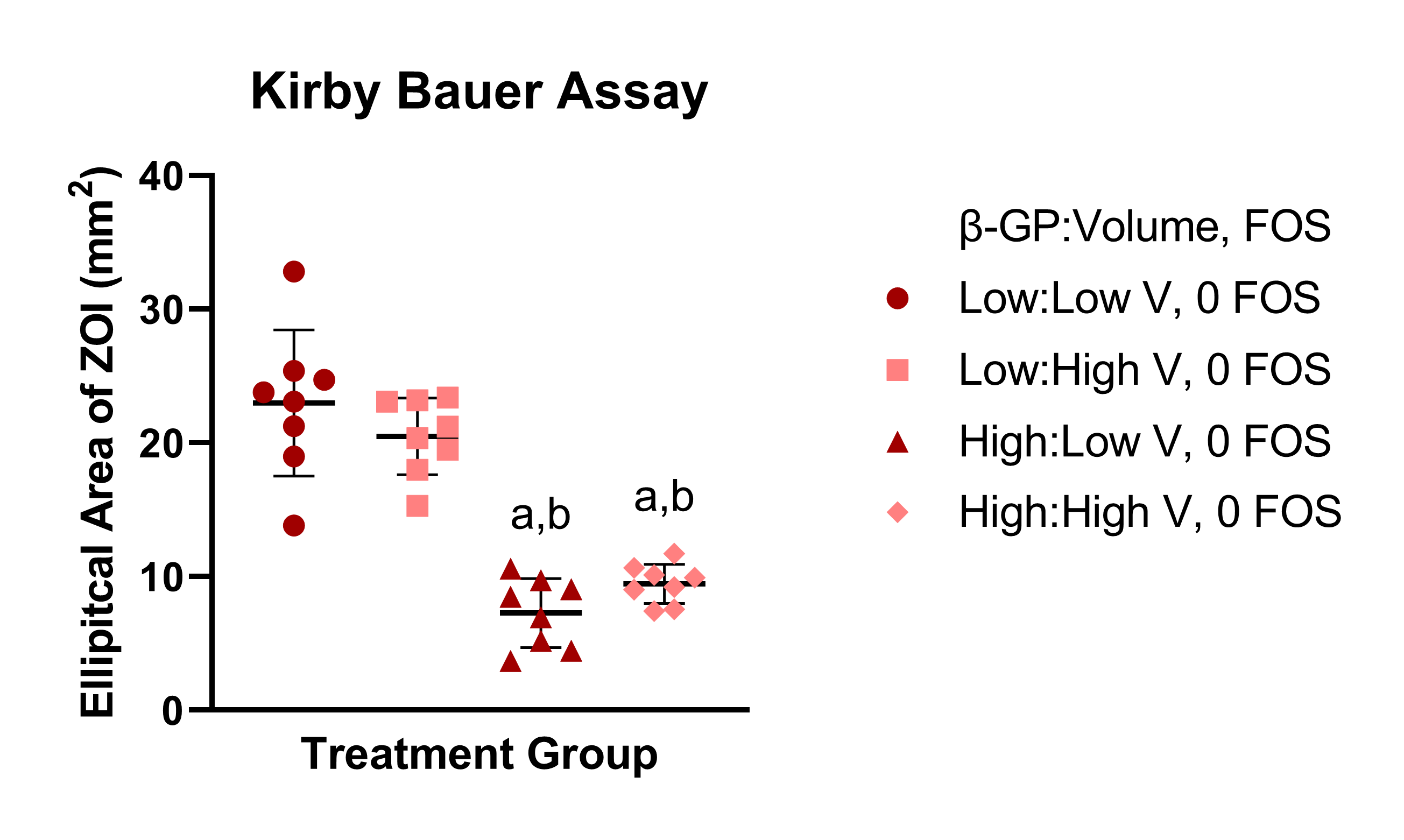

Supplement: Supplementary file 1 [file marinedrugs-19-00144-s001.zip › Supplemental/Figure_S1.tif]

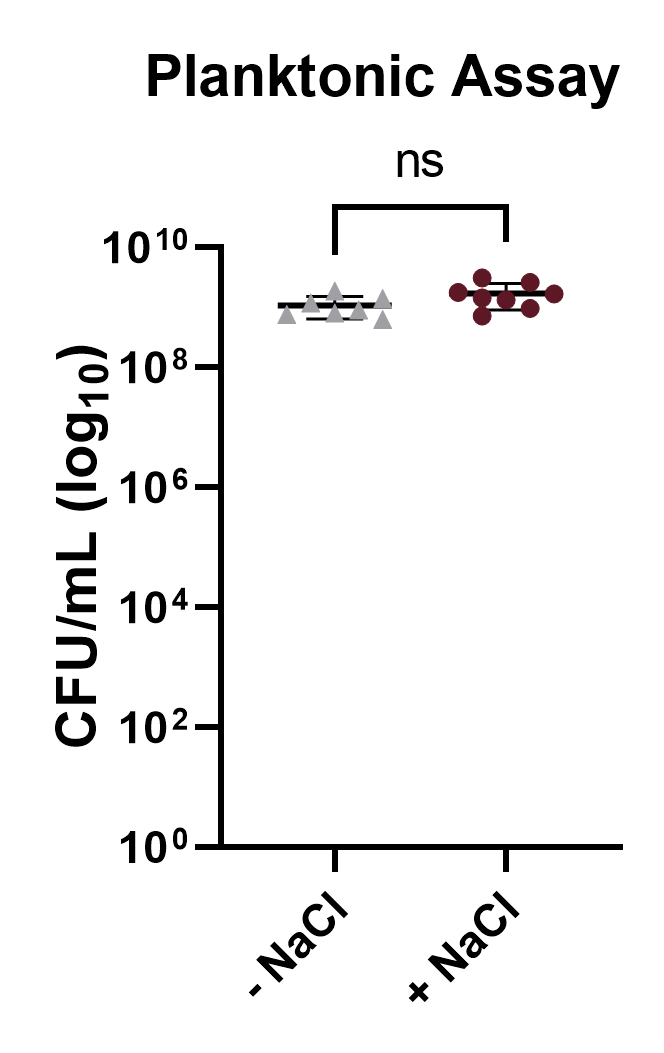

Supplement: Supplementary file 1 [file marinedrugs-19-00144-s001.zip › Supplemental/Figure_S2.tif]

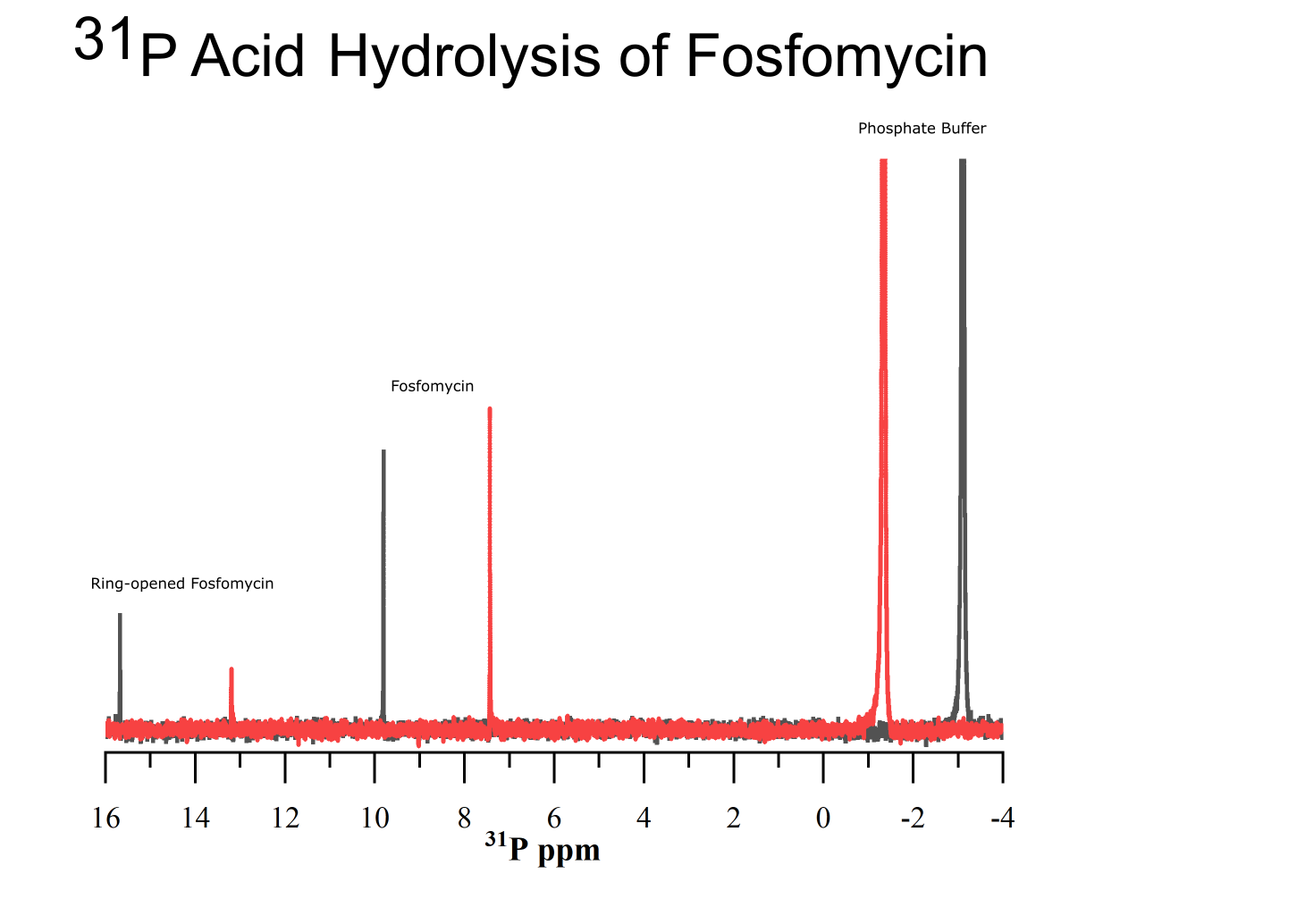

Supplement: Supplementary file 1 [file marinedrugs-19-00144-s001.zip › Supplemental/Figure_S3.png]

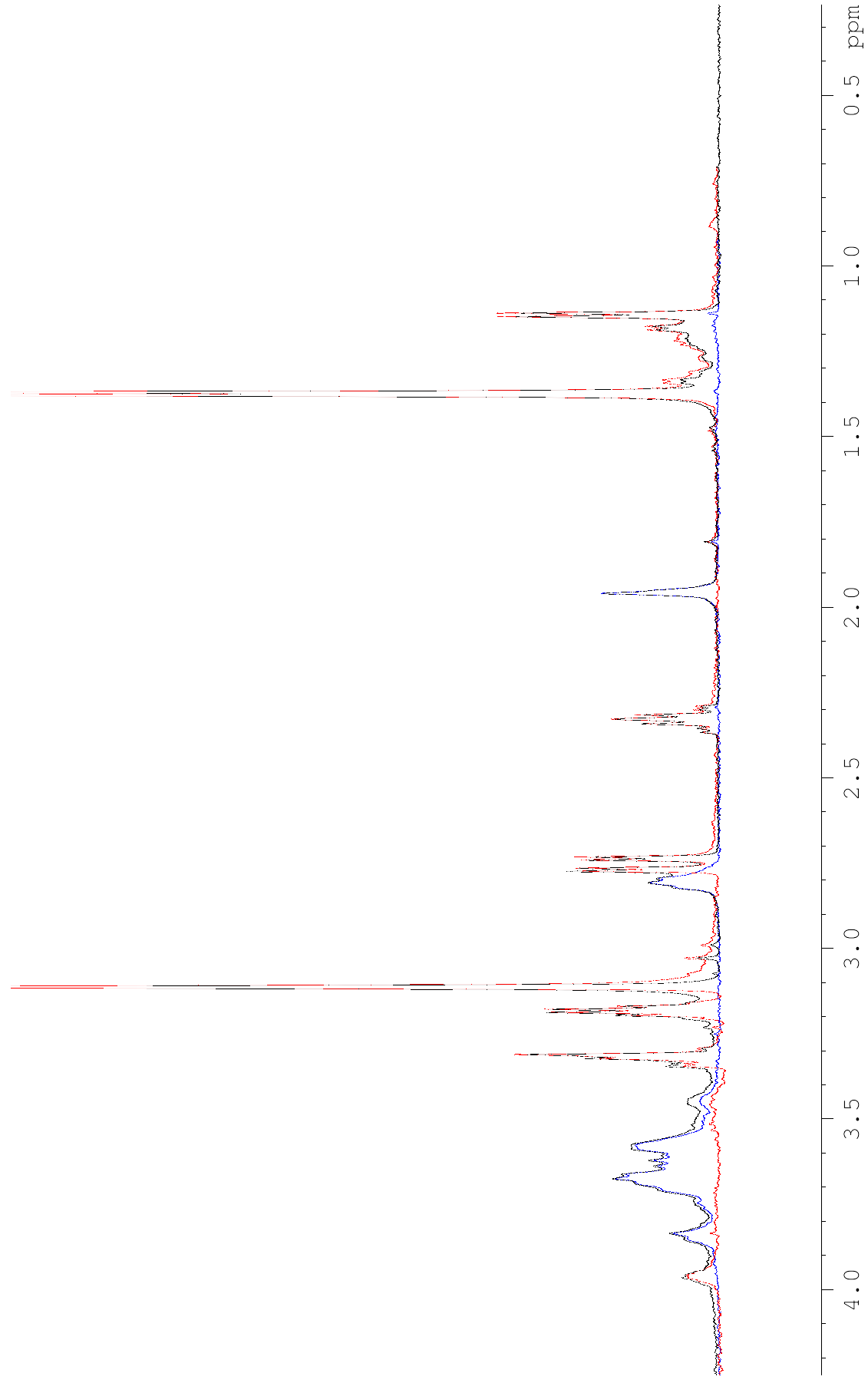

Supplement: Supplementary file 1 [file marinedrugs-19-00144-s001.zip › Supplemental/overlaid.pdf]

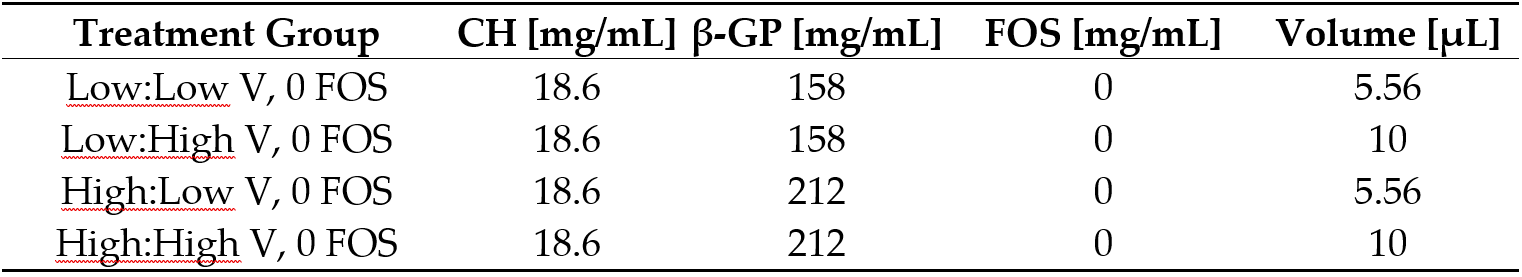

Supplement: Supplementary file 1 [file marinedrugs-19-00144-s001.zip › Supplemental/Table_S1.tif]
